# Atypical Aggressive Hemangioma of Thoracic Vertebrae Associated With Thoracic Myelopathy—A Case Report and Review of the Literature

**DOI:** 10.1155/2024/2307950

**Published:** 2024-08-13

**Authors:** Krishna Timilsina, Sandesh Shrestha, Om Prakash Bhatta, Sushil Paudel, Rajesh Bahadur Lakhey, Rohit Kumar Pokharel

**Affiliations:** ^1^ Pokhara Academy of Health Sciences, Pokhara, Nepal; ^2^ Department of Orthopaedics and Trauma Surgery Tribhuvan University Teaching Hospital, Kathmandu, Nepal; ^3^ Department of Emergency Medicine Nova Hospital, Dhangadhi, Nepal

**Keywords:** aggressive hemangioma, embolization, spinal cord compression, spinal neoplasms, thoracic vertebrae

## Abstract

Aggressive thoracic hemangiomas are rare, benign tumors that extend into the spinal canal and cause neurological symptoms. Delayed diagnosis and treatment, due to a paucity of literature on optimal treatment strategies, can increase morbidity. This case report describes a 19-year-old male patient with aggressive thoracic hemangioma who presented with upper back pain and progressive weakness of the lower extremities. The patient underwent preoperative embolization and sclerotherapy, followed by decompression, posterior instrumentation, and stabilization. The final diagnosis was confirmed by biopsy, and there was a significant improvement in neurology after the surgical intervention. The diagnosis of rare lesions, such as aggressive hemangiomas, requires a high level of clinical suspicion and the assistance of imaging modalities in patients with features of compressive myelopathy. A combination of endovascular and surgical approaches can lead to optimal outcomes.

## 1. Introduction

Vertebral hemangiomas are the most common benign asymptomatic tumors of the spine, occurring in an estimated 10%–12% of the general population. Although less than 1% of these tumors become symptomatic due to extraosseous expansion and the resulting spinal cord compression, they are referred to as aggressive hemangiomas. [1] Vertebral hemangiomas typically occur in the lower thoracic region of the body [2]. It is a great mimicker and can be confused with malignant vertebral tumors and spinal tuberculosis [3, 4]. Diagnosis is based on clinical observations and magnetic resonance imaging (MRI) results confirmed by biopsy. Aggressive vertebral hemangiomas are characterized by the involvement of the entire vertebral body, extension to the neural arch, expanded cortex with indistinct margins, and a soft-tissue mass. These features differentiate between aggressive and benign hemangiomas. An aggressive hemangioma reveals a hyperintense lesion involving the vertebra with the extraosseous extension of the tumor into the spinal canal on MRI. Histologically aggressive hemangiomas typically exhibit increased cellularity, atypical endothelial cells, and a higher number of blood vessels than benign hemangiomas do [1, 5–7]. Although there is no consensus, many treatment modalities are recommended, ranging from embolization-only to en bloc vertebrectomy [8]. Treatment choice largely depends on the clinical presentation of the patient, availability of technology, and preference of the treating physician or institution. We report a case of aggressive hemangioma of the thoracic vertebrae associated with progressive thoracic myelopathy managed with preoperative embolization, followed by spinal cord decompression and posterior instrumentation for stabilization. This case has been reported in line with the SCARE checklist [9].

## 2. Case Presentation

A 19-year-old male presented with dull upper back pain and gradual weakness in the bilateral lower limbs that caused difficulty walking for an 8-month duration. The patient had no history of trauma, fever, weight loss, or loss of appetite. Clinical examination revealed an unsteady wide-based gait with bilateral lower limb spasticity. Exaggerated deep tendon reflexes of the bilateral knee and ankle, positive ankle clonus, and altered sensation below the fourth thoracic (T4) dermatome were present, suggesting thoracic myelopathy. The neurology of the upper extremities was normal. The patient showed no features suggestive of cerebellar lesions.

**Figure 1:**
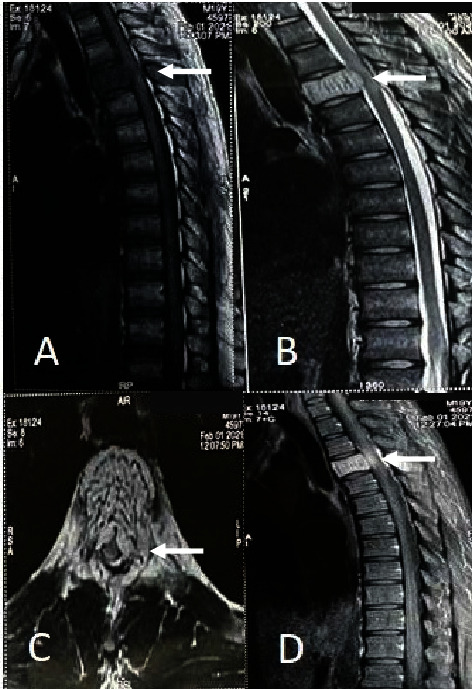
MRI findings. (A) T1 and (B) T2 sagittal images showed hypointense and hyperintense signals in the body and posterior elements, respectively. (C) T2 weighted axial image at T4 vertebrae showing increased signal intensity with cord compression (D) Contrast-enhanced sagittal image showing lesion with soft tissue extension posterior to the body causing almost circumferential compression of the cord.

**Figure 2:**
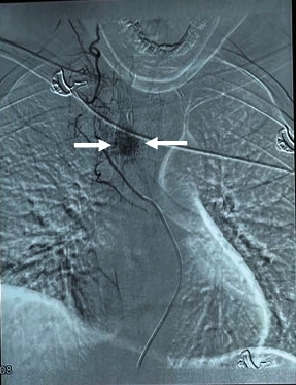
Digital subtraction angiography (DSA) findings. Selected DSA at T3–T4 levels demonstrating abnormal pooling of contrast (white arrow) in T4 vertebral body consistent with a hemangioma.

Plain radiography of the thoracic spine showed thickened vertically oriented trabecula involving the entire T4 vertebral body. MRI of the thoracic spine showed mild enlargement of the T4 vertebral body with a subtle increase in the concavity of the endplate. T1 and T2-weighted images showed hypointense and hyperintense signals in the body and posterior elements, respectively. Gadolinium contrast MRI showed diffuse bright enhancement of lesion with soft tissue extension posterior to the body, causing almost circumferential compression of the cord. The central canal was moderate to severely stenosed (Figure 1). The findings of the MRI were suggestive of atypical (fat-poor) aggressive hemangioma with soft tissue component causing thoracic myelopathy. Spinal angiography revealed a corresponding tumor blush supplied by the right superior intercostal artery at the T4 level (Figure 2). Embolization was performed using polymethyl methacrylate (PMMA) particles to decrease intraoperative bleeding. Because the patient had progressive neurological deficits, he was scheduled for laminectomy and surgical decompression instead of embolization alone. Given the extent of the lesion involving both the vertebral body and posterior elements with epidural extension, a combined embolization and surgery approach was deemed necessary.

**Figure 3:**
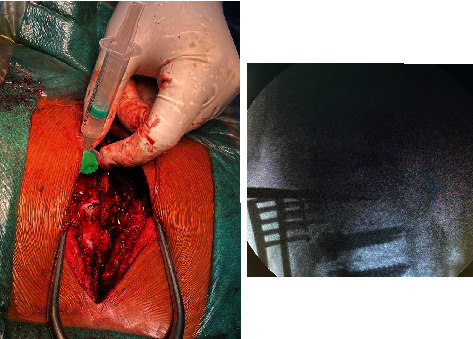
Intraoperative sclerotherapy. Intraoperative picture showing fluoroscopic guided insertion of alcohol (99.9% ethanol) with sodium tetradecyl sulfate on T4 vertebral body.

**Figure 4:**
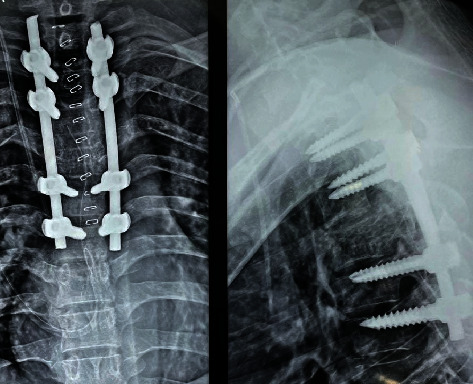
Postoperative X-ray showing posterior instrumentation and stabilization done T2 to T6 levels with pedicle screw fixation at T2, T3, T5, and T6.

**Figure 5:**
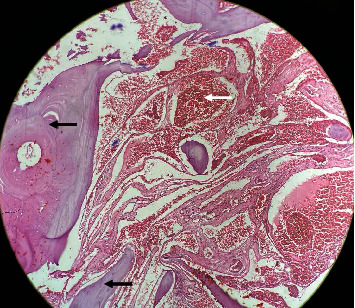
HPE findings suggestive of osseous hemangioma. Histopathological examination (HPE) shows thin-walled blood vessels of various sizes (white arrow) filled with blood and sinuses lined by endothelium between the thickened, vertically oriented trabeculae of bone (black arrow), consistent with osseous hemangioma.

Surgery was planned to decompress the spinal cord, perform a biopsy, and stabilize the spine posteriorly. Pedicle screw fixation with bilateral laminectomy was performed, and as much of the mass as possible in the epidural space was removed and sent for biopsy. Preoperatively, 99.9% ethanol with sodium tetradecyl sulfate was injected into the T4 vertebral body via the transpedicular route to minimize bleeding (Figure 3). Posterior stabilization was performed with long-segment pedicle screw fixation from the T2 to T6 levels (Figure 4). Biopsy revealed features suggestive of aggressive hemangioma (Figure 5).

Postoperatively, the patient showed significant improvement in gait. At the 3 months follow-up visit, the patient had a significant reduction in back pain, gait, and muscle power. At 1-year follow-up, there were no signs of recurrence and the patient showed improvements in gait and muscle power. The patient reported no back pain and was able to perform daily activities.

## 3. Discussion

Vertebral hemangiomas, although generally benign and asymptomatic, can sometimes present aggressively with symptoms, such as dull back pain and progressive neurological deficits. These lesions are usually discovered incidentally during the radiographic evaluation of other conditions [6]. In our patient, who presented with dull aching back pain and gradually increasing neurological symptoms in the lower limbs, the initial clinical diagnosis was Pott's spine because of the endemic nature of tuberculosis in our region. However, a thoracic hemangioma was diagnosed incidentally during imaging to rule out primary or secondary tumors extending into the epidural space.

The majority of vertebral hemangiomas are located in the thoracic spine and are the most common in the fourth to fifth decade of life with a greater female predilection. [1, 5, 6] The term aggressive hemangioma refers to hemangiomas with extraosseous extension or significant osseous expansion [10]. Though some aggressive hemangiomas can be asymptomatic [11], the most common presenting symptom of aggressive hemangioma is back pain, followed by paraparesis or paraplegia [1]. The mechanisms of spinal cord and nerve root compression by hemangiomas include hypertrophy or ballooning of the posterior cortex of the vertebral body, extension through the cortex into the epidural space, and compression fractures [12].

Radiographically, visualizing the upper thoracic vertebra can be challenging, but typical plain radiography images show rarefaction with exaggerated vertical striations creating a ‘corduroy cloth' appearance [5].

Computed tomographic (CT) scans reveal a typical pattern as multiple dots (polka-dot appearance) representing a cross-section of reinforced trabeculae [5, 6].

MRI is more effective in demonstrating extraosseous components. In the T1 image, a typical hemangioma has a high signal due to high lipid content. Atypical hemangiomas have low-fat content and more vascularity and, thus, have low signal intensity in the T1 image. In the T2 image, atypical hemangioma shows a high-intensity signal, greater than on T1, due to its high water content. Contrast-enhanced MRI images show significant enhancement due to the vascularity of the lesion. Thickened trabeculae appear as low-signal areas in both T1 and T2 images, giving the typical appearance of “salt and pepper” [1, 5, 6].

Histologically, vertebral hemangiomas consist of anomalous thin-walled blood vessels and sinuses lined by endothelium, interspersed between thickened, vertically oriented trabeculae of bone. The presence of dilated vascular channels set in a fat stroma is typical [5]. Preoperative digital subtraction angiography is essential for identifying arterial feeders and facilitating treatment planning [13].

There are many treatment options for aggressive vertebral hemangioma [14] ranging from conservative therapy and embolization to total en bloc spondylectomy [15, 16]. Embolization, previously thought to be an adjunct to surgery, nowadays can be used as a definitive treatment. Ethanol vertebroplasty [17], cement vertebroplasty [18], and radiotherapy [7] have been recommended by some authors; and these procedures can be combined with surgery [19]. Radiotherapy alone or following surgery was found to cause total or near-total regression of the tumor [6]. However, repetitive irradiation can cause malignant transformation of the tumor. Some authors have used a combination of treatment modalities: preoperative embolization, total excision, and posterior instrumentation [10, 14, 19]. Preoperative bleeding is a challenge. Preoperative embolization of the feeder vessels [13], careful dissection, and pulse injections of the sclerosing agent into the vertebra [20] are key to minimizing it. The treatment strategy of combined preoperative embolization followed by tumor resection (laminectomy and removal of the tumor from the spinal canal) and posterior spinal stabilization is a safe and effective procedure for treating aggressive vertebral hemangioma. [21, 22]

Comparison of our case with the existing literature highlights the importance of a tailored approach based on the severity of neurological deficits and imaging findings. The reviewed studies and case reports emphasize variability in the presentation and treatment of aggressive vertebral hemangiomas. Commonly, radiotherapy is recommended for less severe cases, whereas surgical intervention is reserved for more significant neurological deficits [7]. Our case aligns with the literature advocating a multimodal approach, particularly for severe presentations. The use of preoperative embolization in our case effectively managed the intraoperative bleeding, a common challenge noted in the literature [6]. Furthermore, the combination of endovascular and surgical techniques appears to optimize outcomes, reduce morbidity, and improve neurological function.

## 4. Conclusion

In cases presenting with symptoms of thoracic myelopathy, rare lesions, such as aggressive hemangiomas, should be considered in the differential diagnosis. A high level of clinical suspicion is facilitated by advanced imaging techniques such as MRI, which can be confirmed by biopsy. This underscores the efficacy of a multidisciplinary approach that combines preoperative embolization, sclerotherapy, and surgical intervention for the optimal treatment of atypical vertebral hemangiomas. Future practice should consider integrating these strategies into standardized treatment protocols to optimize patient outcomes and reduce the morbidity associated with delayed diagnosis and treatment.

## Data Availability

All data underlying the results are available as part of the article.

## Nomenclature

MRI: magnetic resonance imaging
T4: fourth thoracic vertebra
PMMA: polymethyl methacrylate
CT: computed tomography
DSA: digital subtraction angiography
HPE: histopathological examination
